# A Phase 1 Proof of Concept Study Evaluating the Addition of an LSD1 Inhibitor to Nab-Paclitaxel in Advanced or Metastatic Breast Cancer (EPI-PRIMED)

**DOI:** 10.3389/fonc.2022.862427

**Published:** 2022-06-03

**Authors:** Thiru Prasanna, Laeeq Malik, Robert D. McCuaig, Wen Juan Tu, Fan Wu, Pek Siew Lim, Abel H. Y. Tan, Jane E. Dahlstrom, Philip Clingan, Eugene Moylan, Jeremy Chrisp, David Fuller, Sudha Rao, Desmond Yip

**Affiliations:** ^1^ Australian National University (ANU) Medical School, Australian National University, Canberra, ACT, Australia; ^2^ Department of Medical Oncology, The Canberra Hospital, Garran, ACT, Australia; ^3^ Faculty of Science and Technology, University of Canberra, Bruce, ACT, Australia; ^4^ Gene Regulation and Translational Medicine Laboratory, Infection and Inflammation Program, Queensland Institute of Medical Research Berghofer, Brisbane, QLD, Australia; ^5^ Department of Anatomical Pathology, ACT Pathology, The Canberra Hospital, Canberra, ACT, Australia; ^6^ Department of Medical Oncology, Southern Medical Day Care Centre, Wollongong, NSW, Australia; ^7^ Department of Medical Oncology, Liverpool Hospital, Liverpool, NSW, Australia; ^8^ EpiAxis Therapeutics Pty Ltd, Sydney, NSW, Australia

**Keywords:** breast cancer, lysine-specific demethylase 1, cancer stem cells, circulating tumor cells, metastasis

## Abstract

**Objective:**

Lysine-Specific Demethylase-1 (LSD1) is overexpressed in breast cancer cells and facilitate mesenchymal properties which may contribute to therapeutic resistance and cancer progression. The purpose of this study was to investigate the safety of combination, nab-paclitaxel and phenelzine, an irreversible LSD1 inhibitor in patients with metastatic breast cancer (mBC).

**Methods:**

Eligible patients with mBC were treated with nab-paclitaxel (100mg/m^2^) weekly for 3 weeks with one week break in a 28-day cycle. Dose escalation of phenelzine followed the Cumulative Cohort Design and phenelzine treatment commenced from day 2 of first cycle. Eleven patients were screened, and eligible patients were enrolled in cohorts with the dose of phenelzine ranging from 45mg to 90mg.

**Results:**

The Optimum Biological Dose was established at 60mg of phenelzine daily in combination with nab-paclitaxel and considered as the recommended phase 2 dose. Most (95%) of adverse events were grade 1 or 2 with two grade 3 events being diarrhea and neutropenia at 45mg and 60mg phenelzine respectively, with no unexpected toxicity/deaths. Commonly reported toxicities were fatigue (n=4,50%), dizziness (n=6,75%), neutropenia (n=3,37.5%), peripheral neuropathy (n=3,37.5%), diarrhea (n=2,25%), and hallucination (n=2,25%). After a median follow up of 113 weeks, all patients showed disease progression on trial with 4 patients being alive at the time of data cut off, including one patient with triple negative breast cancer. Median progression-free survival was 34 weeks. Significant inhibition of LSD1 and suppression of mesenchymal markers in circulating tumor cells were noted.

**Conclusion:**

Phenelzine in combination with nab-paclitaxel was well tolerated, without any unexpected toxicities in patients with mBC and demonstrated evidence of antitumor activity. For the first time, this proof-of-concept study showed *in-vivo* inhibition of LSD1 suppressed mesenchymal markers, which are known to facilitate generation of cancer stem cells with metastatic potential. **Clinical Trial Registration:** ClinicalTrials.Gov NCT03505528, UTN of U1111-1197-5518.

## Introduction

Breast cancer remains the most prevalent malignancy for women of all ages and is the major cause of cancer mortality in women. Treatment for early stage breast cancer typically includes a combination of local surgery, adjuvant radiation, chemotherapy and endocrine therapy (1). Although breast cancer related mortality has improved over the last two decades, a significant proportion of patients with early stage breast cancer develop metastatic disease after initial curative treatment. Despite advances in systemic therapies with improvement in median survival, many patients (in particularly those with metastatic triple negative breast cancer) will succumb to cancer (2). Cancer stem cells (CSCs) are a small subpopulation of cancer cells found in tumors which are resistant to standard systemic therapy and largely considered to be responsible for tumor progression and metastasis (3). The tumor microenvironment plays a crucial role in exerting selective pressure during the development of CSCs with metastatic properties. The combination of local hypoxia and multiple cytokine pathways is thought to maintain epithelial mesenchymal transition (EMT) and CSCs (4). Furthermore, protein kinases such as protein kinase C theta (PKC-θ), LSD1, and histone deacetylases (HDACs) play a crucial epigenetic role in the initiation and development of CSCs (5, 6). EMT is also thought to be responsible for the generation of circulating tumor cells (CTCs). The phenotype of CTCs may vary between mesenchymal and epithelial status in a given patient (7). High or increasing levels of CTCs are associated with poor prognosis, therefore sequential monitoring of CTC may potentially guide changes in anti-cancer therapy (8) as CTCs with mesenchymal properties are typically resistant to standard systemic therapies and may be crucial in metastasis and thus patient survival.

The taxanes (paclitaxel, docetaxel and nab-paclitaxel) are an important therapeutic class of agents in the standard treatment of early and advanced breast cancer. However, despite providing impressive initial responses, treatment with taxanes is associated with an enrichment of CSCs (6). Boulding et al. demonstrated upregulation of mesenchymal markers like epidermal growth factor receptor (EGFR) and transcription factor SNAI1 in residual MDA-MB-231 cells after treatment with nab-paclitaxel or docetaxel (6). A significant increase in LSD1, especially its nuclear fraction (LSD1-s111p) was noted in these resistant MDA-MB-231 cells. Subsequently, MDA-MB-231 xenograft mice that were treated with the LSD1 inhibitor phenelzine, had reduced tumor growth and the tumor cells isolated showed a downregulation of LSD1 expression and other CSC markers such as cell surface vimentin (CSV), aldehyde dehydrogenase 1 family, member A1 (ALDH1A1) and ATP-binding cassette sub-family B member 5 (ABCB5). The inhibition of LSD1-s111p by phenelzine was shown to occur through binding to both the flavin adenine dinucleotide (FAD) and nuclear REST corepressor 1 (CoREST) domains in contrast to other catalytic LSD1 inhibitors, which do not bind to the CoREST domain (9). Furthermore, LSD1 also seemed to modulate the PD1/PD-L1 axis and regulate T-cell exhaustion transcription factors, which can also impact anti-tumor immunity (10).

LSD1 is over expressed in certain tumor types, correlating with invasion, metastasis and a more aggressive behavior and unfavorable clinical outcome (11–13). Therefore, it was hypothesized that LSD1 inhibition may reprogram and inhibit CTC with mesenchymal phenotype or CSC in patients with metastatic breast cancer, and could potentially reduce tumorigenesis, metastasis and improve survival. Phenelzine, is an approved monoamine oxidase inhibitor (MAOI) typically used for psychiatric indications, but more recently is also recognized as an inhibitor of LSD1 (14). Preclinical data suggest phenelzine inhibited nuclear phosphorylated LSD1 efficiently with corresponding significant histone methylation and CSC inhibition (6).

The objective of this phase 1 study was to determine the maximum tolerated dose (MTD), optimum biological dose (OBD) and to evaluate the safety, and tolerability of phenelzine in combination with nab-paclitaxel in patients with metastatic breast cancer. Cancer cell reprograming was assessed by using a liquid biopsy approach, measuring CTC, a panel of biomarkers and creating a novel phenotypic index, as a proxy pharmacodynamic measure. To the best of our knowledge, this Phase 1 clinical trial is the first published clinical trial to use LSD1 inhibition in combination with chemotherapy (in this case nab-paclitaxel).

## Materials and Methods

### Patient Eligibility

Patients with a histologically confirmed locally advanced (inoperable) or metastatic breast cancer who were planned to be treated with nab-paclitaxel were enrolled. Other eligibility criteria included: age ≥18 years; Eastern Cooperative Oncology Group (EGOG) performance status of <2; previous chemotherapy >3 weeks before the first dose; adequate bone marrow function (absolute neutrophil count >1,500/mm3, platelet count >100,000/mm3); adequate hepatic function (bilirubin <1.5 times upper limit of normal, AST and ALT <2 times upper limit of normal or <5 times ULN if liver metastases present); and adequate renal function (serum creatinine <1.5× upper limit of normal). Patients were permitted to have had prior chemotherapy (except nab-paclitaxel), radiation therapy, or biological therapy. Exclusion criteria included untreated, uncontrolled leptomeningeal or brain metastases, concurrent use of sympathomimetic agents or MAOI inhibitors or other central nervous system depressants.

Patients with HER2-positive metastatic breast cancer, progressive brain metastases, uncontrolled systemic hypertension, and clinically significant anemia were excluded. Due to the mechanism of action of phenelzine, and to avoid excessive associated toxicity, patients who were on MAOI, serotoninergic and sympathomimetic agents and certain pain management agents (buspirone, pethidine, tramadol, dextromethorphan, fentanyl and/or methadone) were also excluded. A full list of inclusion/exclusion criteria is provided in **Supplementary Table 1**.

### Study Design and Conduct

This open label Phase I study was conducted in three cancer centers in Australia with approvals from all relevant institutional Human Research Ethics Committees. (ACT ETH.10.16.218) All participating patients signed an informed consent document in accordance with the Declaration of Helsinki. The trial was registered on the Australia and New Zealand, and American Clinical Trials Registers (ACTRN12617000943347; ClinicalTrials.Gov NCT03505528) and was assigned a UTN of U1111-1197-5518. The study was conducted in patients with locally advanced inoperable or mBC to assess the safety/tolerability, pharmacokinetics, and antitumor activity of the phenelzine/nab-paclitaxel combination therapy over three 28-day cycles.

**Figure 1:**
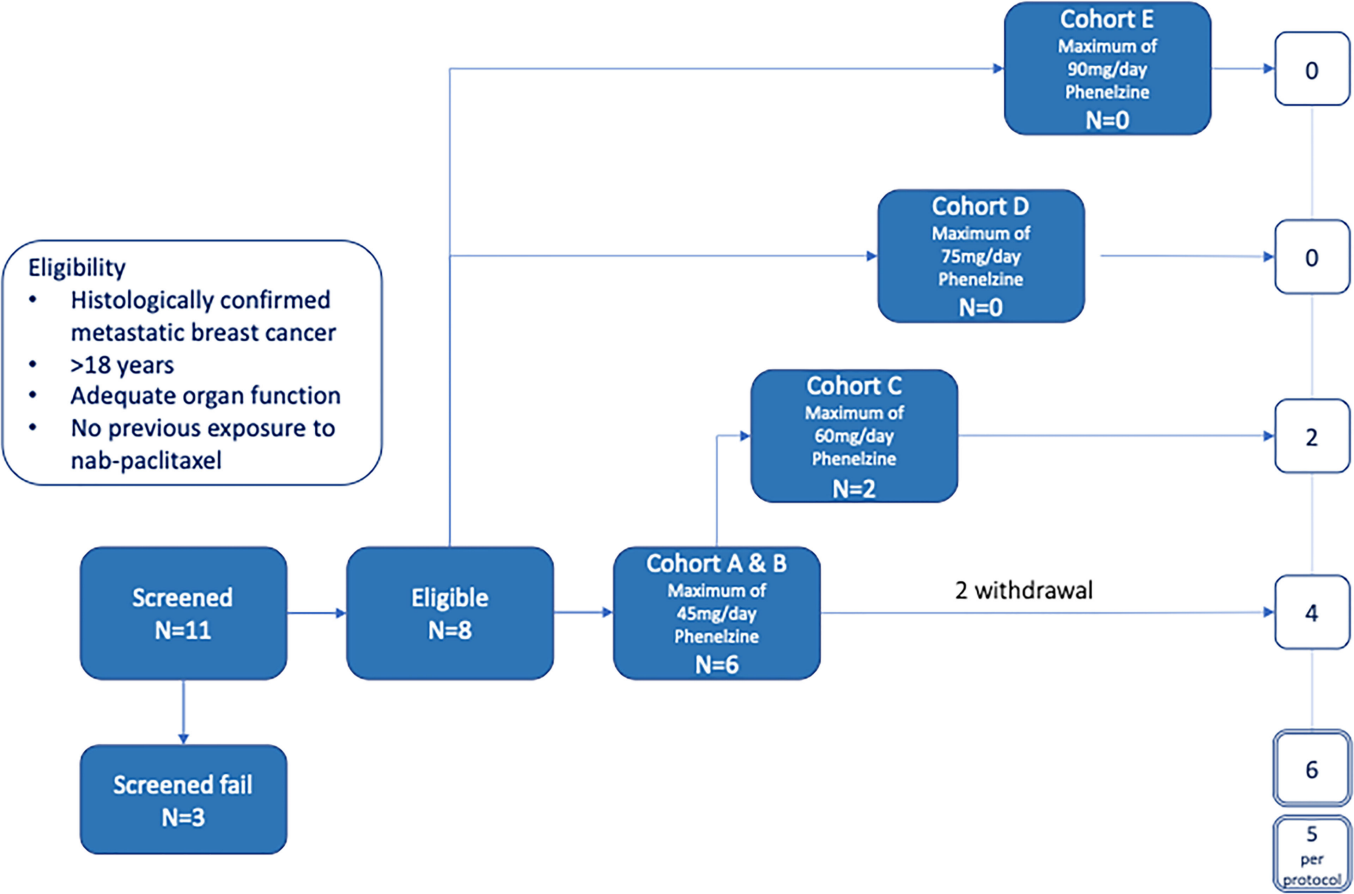
Study design.

The study was designed to have 5 cohorts – an initial sentinel cohort of phenelzine titrated from 15 to 45 mg daily, followed by cohorts of 45, 60, 75 and 90mg phenelzine total daily dose (administered in divided equal aliquots three times per day) (**Figure 1**). Phenelzine has a well-established safety profile for the treatment of depression with FDA recommended starting dose being 15mg three times daily. Therefore, 15mg once daily was chosen as a starting dose in combination with nab-paclitaxel held at a constant dose of 100mg/m^2^. Phenelzine treatment commenced in cycle 1, (day 2 to day 84) and dose was escalated in the absence of any dose limiting toxicity (DLT). Dose escalation of phenelzine followed the Cumulative Cohort Design (CCD) of Ivanova et al. (15), using a target toxicity level (probability of a DLT at MTD of 0.3 (15). Dose-escalation decisions were agreed upon by the Investigators and the study Sponsor based on an assessment of DLTs, adverse events (AEs), and laboratory data during a DLT window of day 2 to day 56. When the dose of phenelzine was more than 45mg/day, treatment was commenced at 45mg/day and escalated to the target dose with weekly 15mg increments. Dose reductions were instituted when a patient experienced grade 3 or 4 adverse event or a DLT. A maximum of one dose reduction was allowed for nab-paclitaxel and phenelzine after cycle 2. Patients who were benefiting from treatment were allowed to continue study treatments until they experienced disease progression or unacceptable toxicity.

### Treatment

The study comprised a maximum of 3 x 28 day cycles of combination nab-paclitaxel (Abraxane, Specialised Therapeutics) and phenelzine (Nardil, Link Medical Products Pty Ltd). Patients received nab-paclitaxel 100mg/m^2^ as an intravenous infusion over a period of 30 mins once a week for three consecutive weeks, with one week break before commencing the next cycle. Phenelzine treatment commenced on Day 2 of Cycle 1 and continued through to the conclusion of the study. Dose modifications or delays of nab-paclitaxel chemotherapy were executed according to standard clinical practice. The highest planned phenelzine dose was 90mg daily (30mg tds) corresponding to the maximum recommended dose for treatment of depression (16).

### Purpose and Assessments

The primary objective was to determine the safety and tolerability of the phenelzine and nab-paclitaxel combination and to define the MTD and OBD. The secondary objectives included an assessment of antitumor activity and exploration of relationships between phenelzine dose and CTC/CSC burden, and dynamic real time assessment of the immune response and various cell surface markers associated with epithelial to mesenchymal transition (EMT). CTCs were enriched from liquid biopsies after CD45 depletion (15162, Stemcell Technologies) and removal of red blood cells as previously described (6, 10). CD45-negative enriched cells were stained with different antibodies against proteins related to EMT/CSC and sorted using DEPArray (17). CTC enrichment and enumeration was conducted at the beginning of each cycle (every 28 days) and at the end of study. The OBD was defined as the dose at which optimal LSD1 inhibition and CTC depletion was achieved.

Safety was assessed by physical examination, ECG, clinical laboratory assessments (hematology, biochemistry) for all patients entering the trial and receiving at least one dose of phenelzine. Any AE that occurred after start of study treatment or worsened during study treatment was defined as a treatment-emergent adverse event (TEAE). The severity of adverse events was graded using categories of mild, moderate and severe with reference to CTCAE v4.03 and DLTs were assessed until the end of cycle 3. DLTs were prospectively defined to included grade 3 febrile neutropenia, > grade 2 peripheral neuropathy and any serious adverse events (SAE). (**Supplementary Table 2**). The MTD was defined as the dose of phenelzine at which 30% of patients experienced a DLT. Measures of antitumor effect were, objective response rate, complete response [CR], partial response [PR], disease control rate [CR+PR+stable disease/SD], progression-free survival (PFS) and overall survival (OS). Response to treatment was assessed using Response Evaluation Criteria in Solid Tumors (RECIST) Version 1.1 (18).

### Immunofluorescence Staining and Microscopy

Immunofluorescence imaging and analysis was carried out using previously established and optimized protocols (6, 10). Cells were stained with appropriate primary and secondary antibodies. Protein targets were localized by confocal laser scanning microscopy. Single 0.5 μm sections were obtained using a Leica DMI8 microscope running LAX software using a 100x oil immersion lens. The final image was obtained by averaging four sequential images of the same section. Digital images were analyzed using ImageJ-Fiji software (ImageJ, NIH, Bethesda, MD) to determine either the nuclear, cytoplasmic or whole cell fluorescence. The Two-Way ANOVA test (GraphPad Prism, GraphPad 178 Software, San Diego, CA) was used to determine significant differences between datasets.

### Immunofluorescence Analysis: Phenotypic Score

Before the study commenced, it was hypothesized that treatment with nab-paclitaxel and phenelzine would result in a phenotypic change in CTCs. That change was hypothesized to be associated with decreased expression of the markers CSV, EGFR, FOXQ1, LSD1, PD-L1 and SNAI1, and increased expression of the markers H3K9me2 and H3K4me2. The CSV and PD-L1 were measured in cytoplasm. For each cellular localisation, the score consisted of the difference between the average of the markers hypothesised to decrease, and the average of the markers hypothesised to increase. The phenotypic score was plotted against visit and dose, and analysed as above using a mixed effect model with fixed effects for visit and dose and random patient effect (**Box 1**).

Box 1 **Phenotypic Score Definition for Time Trend Analysis; Component Score.**

Cytoplasm (CSV + EGFR + FOXQ1 + LSD1 + PD-L1 + SNAI1)/6 -(H3k9me2 + H3K4me2)/2

Nucleus (EGFR + FOXQ1 + LSD1 + SNAI1)/4 -(H3K9me2 + H3K4me2)/2

Whole Cell (EGFR + FOXQ1 + LSD1 + SNAI1)/4 -(H3K9me2 + H3K4me2)/2

The marker values in the expressions shown above represent the mean of all CTCs for each patient and visit. Scores for each component were plotted against visit (coded numerically-**Box 1**) after treatment, by phenelzine target.

### Statistical Analyses

The planned sample size was for between 10-20 patients, and it was not based on a formal calculation due to the experimental nature of a Phase 1 trial. Safety parameters were analyzed using descriptive statistics. The data cut-off date for the analyses presented in this report is April 2021. Statistical analysis of clinical, biomarker and phenotypic index was performed by Emphron Informatics using R programming language. Time trends were calculated for each marker and cellular localisation separately, using a linear mixed effects model with fixed effects for visit (in sequential order), dose, and their interaction and random patient effect; p-values were corrected to account for multiple testing using false discovery rate corrections (19, 20).

## Results

### Study Disposition

A total of 11 patients with locally advanced inoperable or mBC provided written informed consent to participate in the EPI-PRIMED study. Eight patients received at least one dose of phenelzine and nab-paclitaxel, while 3 patients failed screening. The median patient age was 59.0 years (range 35-73) with median age of initial breast cancer diagnosis 44.5 years (range 34-62) (**Table 1**). Three patients had triple negative breast cancer (TNBC) and five patients had estrogen receptor positive (ER+) mBC. All 8 participants enrolled into study received at least 1 dose of both investigational products; however, only 5 of the 8 participants completed the study as per protocol.

**Table 1:** Demographic data.

Participants	Total number	Phenelzine = 45 mg/day	Phenelzine = 60 mg/day
		N = 6	N = 2
**Age of Breast Cancer Diagnosis**	Years, Mean (SD)	45.0 (12.5)	57.5 (9.2)
**Age at Study Enrolment**	Years, Mean (SD)	52.0 (12.8)	67.0 (8.5)
**Phenotype:**			
** MBC Population**	N (%)	3 (50)	2 (100)
** TNBC Population**	N (%)	3 (50)	0 (0)
**Ethnicity:**			
** ATSI**	N (%)	2 (33.3)	0 (0)
** Caucasian**	N (%)	4 (66.6)	2 (100)
** Body Mass Index (BMI)**	Kg/m^2^, Mean (SD)	33.5 (8.4)	33.0 (2.3)
** Recurrent MBC**	N (%)	6 (100)	2 (100)
**Site of metastasis:**	N (%)		
** Liver**		1 (16.6)	0 (0)
** Lung**		3 (50)	0 (0)
** Bone**		2 (33.3)	2 (100)
** CNS**		1 (16.6)	0 (0)
** Other**		2 (33.3)	0 (0)
**Number of lines of therapy for MBC**	N (%)	3 (50%)	1 (50)
**Adjuvant therapy:**			
** Endocrine therapy**		1 (16.6)	2 (100)
** Chemotherapy**		2 (33.3)	2 (100)

### Dose Escalation and Safety

AEs were recorded from the time of the first study dose to the End of Study Visit with a total of 40 TEAEs reported by 8/8 (100%) participants. The most commonly reported events were consistent with the known safety profiles of nab-paclitaxel and phenelzine and included dizziness (including postural dizziness) (n=6), fatigue (n=4), decreased neutrophils (n=3), neuropathy (including peripheral neuropathy) (n=3), diarrhea (n=2) and hallucinations (n=2). All other AEs were single reports. The majority (95%) of TEAEs were either grade 1 or 2. (**Table 2**) There were no treatment related deaths and no events were reported to be life threatening. Two grade 3 events (2/40, 5%) were reported. The first event was an event of neutropenia in a patient receiving 60mg phenelzine leading to a 1-week delay of nab-paclitaxel treatment. The second event was the only SAE reported during the study and occurred in a patient treated with 45mg phenelzine. This was a prolonged episode of diarrhea resulting in hospitalization (and was classified as a DLT) and resulted in study withdrawal. This patient fully recovered to baseline in 6 days. Another patient experienced lightheadedness/dizziness at the start of Cycle 3 and the phenelzine dose was reduced to 30mg, however the patient subsequently experienced hallucinations and withdrew consent later in cycle 3. These neurological symptoms were considered to be related to phenelzine and the patient fully recovered after ceasing study treatments. One patient in the 60mg cohort experienced increasing confusion and mental fogginess. Phenelzine was ceased for 10 days and then resumed at 45mg for the remainder of the study without further incident.

**Table 2:** Treatment-emergent AEs in the safety analysis according to CTCAE grade.

	All reported AEs (n = 40)		Phenelzine 45mg (n = 22)		Phenelzine 60mg (n = 18)	
	Any grade n (%)	Grade >3 n (%)	Any graden (%)	Grade >3 n (%)	Any graden (%)	Grade >3 n (%)
Patients with any AEs	40 (100)	2 (5%)	22 (100)	1 (4.5)	18 (100)	1 (5.5)
Dizziness/ light headedness	9 (22.5)	0	6 (27)	0	3 (16.6)	0
Hallucination/ confusion	8 (20)	0	2 (9)	0	6 (33)	0
Fatigue	5 (12.5)	0	3 (13.6)	0	2 (11)	0
Neuropathy	3 (7.5)	0	3 (13.6)	0	0	0
Neutropenia	3 (7.5)	1 (2.5)	2 (9)	0	1 (5.5)	1 (5.5)
Diarrhea	3 (7.5)	1 (2.5)	2 (9)	1(4.5)	0	0
Nausea	2 (5)	0	1 (4.5)	0	1 (5.5)	0
Peripheral edema	2 (5)	0	0	0	2 (11)	0
skin rash	1 (2.5)	0	0	0	1 (5.5)	0
weight gain	1 (2.5)	0	0	0	1 (5.5)	0
Weight loss	1 (2.5)	0	0	0	1 (5.5)	0
Deranged LFT	1 (2.5)	0	1 (4.5)	0	0	0
QT prolongation	1 (2.5)	0	1 (4.5)	0	0	0

A reduction in CTC count was seen at both the 45mg and 60mg dose of phenelzine, however CTC count at the commencement of treatment was highly variable and made interpretation difficult. However, phenelzine at 60mg inhibited LSD1 significantly more than at 45mg with an associated increased reduction in mesenchymal phenotype. The study was terminated early at 60mg of phenelzine based on the extent of the observed LSD1 and CTC inhibition, which indicated “proof of biological principle” had been established. In consequence an MTD was not established and, per protocol, 60mg of phenelzine was declared as OBD and recommended phase 2 dose (RP2D).

### Efficacy Analysis

Efficacy was included as a secondary objective in this clinical trial *via* RECIST 1.1 response criteria. Limited efficacy data are available as the majority (75%) of enrolled subjects did not have measurable lesions at baseline, with only two (2/8, 25%) patients assessable for end of study RECIST response. Of these two patients, one had stable disease and one had progressive disease at the end of the study. After a median follow up of 113 weeks, 4/8 (50%) patients were alive with a median PFS of 34 weeks (range 8-88 weeks). Survival for the TNBC patients (n=3) was 57 and 76 weeks, while a 3^rd^ patient with TNBC was still alive (149 weeks) on further treatment at the time of data cut off of April 2021.

### Biomarker Analysis

**Figure 2:**
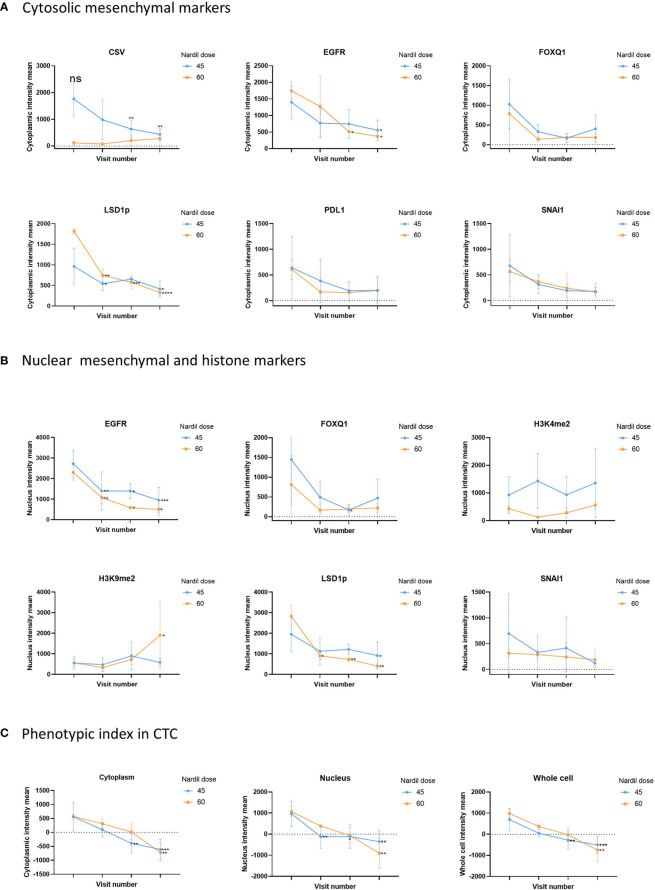
Analysis of CTC mesenchymal markers during the study. Analysis of CTC mesenchymal marker and LSD1 in metastatic breast cancer patient derived CTCs. Figure shows differential changes between phenelzine 45mg (blue) and 60mg (Red). CTCs were isolated at baseline, cycle 1 (Day 29), 2 (Day 57) or cycle 3 (Day 85 end of study). **(A)** Shows changes in mean cytoplasmic intensity of CSV, LSD1s111p (LSD1p), EGFR, FOXQ1, PD-L1 or SNAI1 during the course of trial. **(B)** Shows changes in nuclear intensity of H3K9me2, H3K4me2, LSD1s111p, EGFR, FOX1Q, or SNAI1. **(C)** Phenotypic index, measuring the phenotypic change in the patients CTCs was calculated as described in the methods for the cytoplasmic, nuclear and whole cell scores. This score was then plotted for cycle 1 to 3 compared to baseline readings. Significant differences were determined with a two-way ANOVA with multiple comparisons to baseline and are indicated, *<0.05, **<0.01, ***<0.001, ****<0.001, ns, not significant.

In this clinical trial the effect of phenelzine treatment on a number of mesenchymal markers including CSV, EGFR, FOXQ1, SNAI1 and PD-L1 were assessed using patient derived circulating tumor cells (CTCs) from liquid biopsies taken at baseline and at 29, 57 and 85 days post start of phenelzine therapy. Overall, treatment with phenelzine displayed a trend of decreased cytoplasmic expression of all mesenchymal markers: CSV, EGFR, FOXQ1, PD-L1 and SNAI1 (**Figure 2**), however, only CSV and EGFR reduction were statistically significant. Small number of patients in the 60mg cohort may have contributed to this. LSD1 cytoplasmic expression was significantly reduced in both cohorts with a stronger effect observed in the 60mg cohort (**Figure 2**).

Nuclear expression of SNAI1, FOXQ1 and EGFR reduced over time during the course of treatment with phenelzine (**Figure 2**). Phosphorylated LSD1 (LSD1p) nuclear expression was significantly inhibited in both cohorts with more reduction seen with 60mg of phenelzine. The percentage change of expression relative to baseline was calculated for LSD1p for whole cell, cytoplasmic and nuclear expression as determined by high resolution fluorescent microscopy. Treatment with phenelzine reduced LSD1p expression globally, but most significant inhibition was seen in nuclear compartment compared to cytosolic compartment (**Figure 2**). Overall, expression was lowest at Day 85 at end of study for both the cytoplasmic and nuclear compartments for LSD1 and the mesenchymal markers.

Consistent with reduction of LSD1 expression, H3K4me2 and H3K9me2 nuclear signals (the target histones for LSD1 mediated demethylation) increased over time. The H3K9me2 increment was significantly higher after treatment with 60mg compared with 45mg (**Figure 2**). Whole cell fluorescent signal analysis revealed a similar trend to the cytoplasmic or nuclear compartments with strongest effects seen on EGFR expression and LSD1p expression.

**Figure 3:**
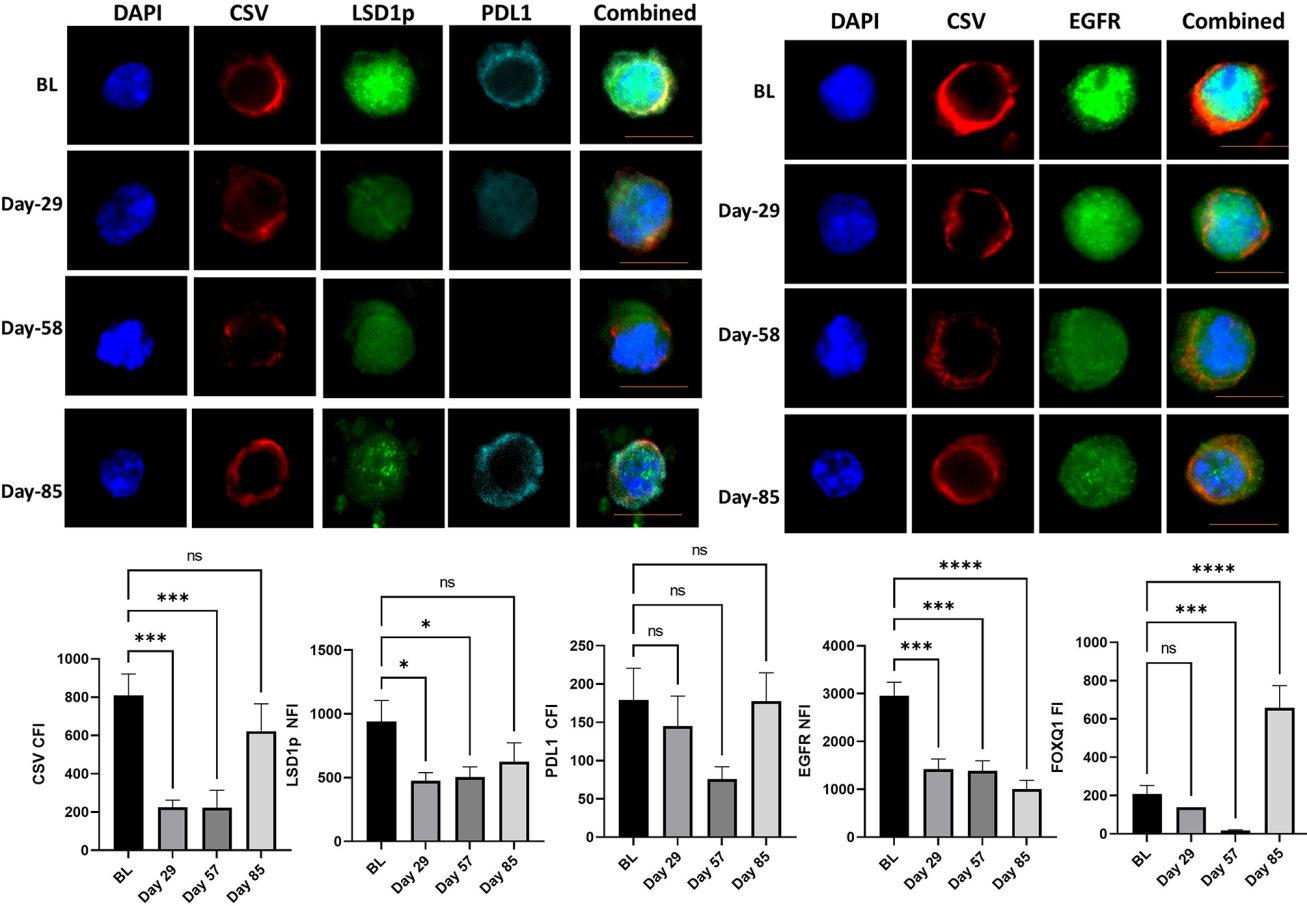
CTC analysis of the patient with triple negative breast cancer with longest survival. The mean cytoplasmic fluorescent intensity (CFI) or the mean nuclear fluorescent intensity (NFI) of mesenchymal markers and PD-L1 were measured in CTC of the patients with triple negative breast cancer with longest survival was. Graphs plot the CFI or NFI mean for Cycle 1 to 3 compared to baseline readings. Significant differences were determined with a two-way ANOVA with multiple comparisons to baseline and are indicated, *<0.05, ***<0.001, ****<0.001, ns, not significant. Example images of CSV, LSD1p and PD-L1 or CSV and EGFR are shown for each time point with the scale bar in orange equal 1 micron.

One patient with TNBC, was still alive at 149 weeks. This patient has now progressed and has received further systemic therapy. CTC analysis of this patient showed significant downregulation of mesenchymal markers, especially at day 57. The most notable changes for this patient were seen in EGFR throughout the trial, while expression of CSV, FOXQ1 and PD-L1 increased at day 85, however, it should be noted number of CTC obtained at day 85 was quite small (**Figure 3**).

### Phenotypic Score in Metastatic Breast Cancer Patient Derived CTCs

To further probe the change in the mesenchymal signature of the CTCs, a “phenotypic score” (**Box 1**) was used to calculate the change. This score is based on the fluorescent intensity of the mesenchymal markers (CSV, PD-L1, FOXQ1, EGFR, SNAI1), the drug target, LSD1, and the histone marks (H3K4me2, H3K9me2), (**Figure 2C**). A reduction of phenotypic score indicates a reduction in the overall mesenchymal signature of the CTC. Phenelzine treatment at either the 45 or 60mg doses significantly abrogated the phenotypic score in cytosolic, nuclear and whole cell compartment.

### Discussion

The primary objective of this clinical trial was to assess the safety of the combination use of nab-paclitaxel with phenelzine over 3 treatment cycles. Nab-paclitaxel was chosen as the preferred chemotherapeutic agent as its administration does not require any concomitant steroids which may interfere with any potential immunomodulatory effects of LSD1 inhibition. As noted previously, both phenelzine and nab-paclitaxel are approved agents with well-defined safety profiles. The safety data reported from this combination study of nab-paclitaxel and phenelzine were consistent with the known safety profiles of each single agent. The majority of TEAEs were either grade 1 or 2 (95%). Two subjects withdrew due to unacceptable neurological side effects attributable to phenelzine. There were no deaths, and no events were reported to be life threatening. The OBD for phenelzine was determined as 60mg daily in combination with nab-paclitaxel 100mg/m^2^.

Analysis of CTC count was difficult due to low numbers; however, a gradual reduction was seen in CTC count with time during the treatment with phenelzine and nab-paclitaxel compared to pre-treatment level (**Supplementary Figure 1**). Although not all studies are consistent, a number of reports (including a meta-analysis) have reported the prognostic value of CTC measurement in metastatic BC patients (8, 21–23). Generation of CTC were thought to be secondary to EMT and responsible for metastasis. CTC in a given patient may consist of both mesenchymal and epithelial phenotypes depending on the tumor microenvironment and status of EMT and Mesenchymal Epithelial Transition (MET) (7). CTC with mesenchymal phenotype may be resistant to standard anticancer therapy and more important for metastasis (24).

Anticancer properties of phenelzine have been previously demonstrated in other solid organ cancers (25). Phenelzine inhibited prostate cancer cells *via* inhibition of monoamine oxidase A (MAOA) which is an androgen regulated enzyme that may facilitate growth of PTEN-dependent prostate cancer (26). Importantly, Prusevich et al. have demonstrated phenelzine is a potent irreversible LSD1 inhibitor, a finding confirmed by other authors (6, 14). Given the critical role nuclear LSD1 in EMT and maintaining mesenchymal phenotype, phenelzine was used to provide LSD1 inhibition in this study. Consistently, combination treatment resulted in progressive suppression of whole cell, and more importantly, nuclear LSD1 expression in CTC with a higher reduction seen with 60mg compared to 45mg of phenelzine treatment.

For the first time, this study shows in humans, dynamic inhibition of LSD1 in breast cancer CTC with associated reduction in expression of mesenchymal markers such as EGFR, FOXQ1, CSV and SNAI1 (6, 27). Despite difficulties in enumeration of CTC, a clear phenotypic transformation of CTC was noticed from more aggressive mesenchymal CTC to epithelial type CTC. Previous studies have shown overexpression of mesenchymal markers EGFR, CSV and SNAI1 after treatment with nab-paclitaxel in breast cancer cell lines (6). This study provides evidence of *in-vivo* suppression of LSD1 by phenelzine and subsequent downregulation of mesenchymal markers (**Figure 2**). The association between LSD1 and PD-L1 expression is not well established with some studies suggesting potential overexpression of PD-L1 with LSD1 inhibition, however in this study PD-L1 expression reduced with time during the treatment with phenelzine and warrants further exploration (28, 29). LSD1 inhibition has been shown to induce a number of other off-target effects in addition to the effect on histones. LSD1 may interact with non-histone proteins like p53, DNMT1 and others. It may also play a role in DNA damage repair mechanism *via* the Retinoblastoma gene 1 (RB1) and related proteins like 53BP1 (30, 31). This study investigated the *in-vivo* epigenetic and mesenchymal modification associated with LSD1 inhibition. However, further molecular characterizations are required in future studies to fully understand the role of LSD1 in the underlying complex molecular pathways.

Prognosis of advanced TNBC is poor with limited development of novel targeted therapies. Expected median OS is approximately less than 18 months. More recently, a PD-L1 inhibitor atezolizumab in combination with nab-paclitaxel has shown modest improvement in PFS (7.2 v 5 months) and OS (21 v 17 months) compared to nab-paclitaxel alone (32). Even though direct comparison of trials is not feasible, an acceptable median PFS of 8.5 months was noted in this study, with 4 patients alive at the time of reporting, including one patient with TNBC. Two patients had stable disease on their subsequent systemic therapy. Out of 4 patients with TNBC, PFS of two patients were 14 and 19 months with the third patient still alive at 37 months on subsequent lines of treatment. However, we note that efficacy assessment in our study was limited due to small numbers and lack of measurable disease in most patients in the study.

Increasing evidence suggest that CSC play an important role in metastasis (33). Presence of distant metastasis is the single most important adverse prognostic factor in invasive breast cancer and CSC are considered responsible for metastasis initiation. CSC are resistant to most standard chemotherapy and radiation. Preclinical studies have shown they are enriched after treatment with nab-paclitaxel (6). Others have demonstrated enrichment of the CSC sub-population after neoadjuvant chemotherapy for breast cancer (34). Consistently, patients with residual cancer after neoadjuvant chemotherapy have poor survival compared to those who achieve complete pathological response (35). Furthermore, in the metastatic setting, even though RECIST criteria are used to assess the efficacy of anti-cancer treatment, tumor regression (reduction in non-CSC population) is not always associated with improved survival (36). Therefore, we hypothesized that depletion of CSC like cells are crucial to improve survival.

LSD1 inhibition with phenelzine has been shown to inhibit CSC in breast cancer cell lines and inhibited tumor growth xenograft models significantly in combination with nab-paclitaxel compared to nab-paclitaxel alone (6). In this trial, we demonstrated for the first time in the clinical setting, that combined LSD1 inhibition along with standard chemotherapy is a feasible, safe approach with dynamic real time measurement in liquid biopsies showing reduction in nuclear LSD1 in CTCs. More importantly, LSD1 depletion inhibited CTC with mesenchymal phenotype and induced a phenotypic change in CTC population from an aggressive mesenchymal CTC to a more epithelial phenotype, which is considered to possess less metastatic potential. Furthermore, high stemness features are associated with high PD-L1 expression and may confer resistance to anti PD1/PD-L1 therapies. Serial CTC obtained from the patient with TNBC who was still alive at 37 months exhibited significant reduction in mesenchymal markers along with reduction in PD-L1 expression during the trial. This suggests that eliminating CTC with mesenchymal properties (or CSC) may be more crucial in preventing metastasis and prolonging survival (**Figure 3**).

This phase 1 study had several limitations, including a small number of patients, lack of RECIST evaluable data in number of patients and lack of independent confirmation of RECIST 1.1 evaluation. Furthermore, the LSD1 inhibitor, phenelzine was not continued beyond the defined duration in the protocol (84 days), with the exception of one patient. However, CTC analysis showed reduction in nuclear LSD1 and CTC number were progressively lower with time. Given epigenetic changes are potentially reversible, a longer duration of treatment with phenelzine may have achieved longer duration of disease control.

This proof-of-concept study showed phenelzine mediated LSD1 inhibition in combination with nab-paclitaxel (100mg/m^2^) had an acceptable safety profile with a signal of antitumor activity and established an RP2D for future studies. To best of our knowledge this is the first study demonstrating LSD1 inhibition in breast cancer resulting in depletion of CTC with stemness features and a phenotypic switch from mesenchymal to more epithelial CTC in patients with metastatic breast cancer. Furthermore, this study shows the feasibility of real time dynamic measurement and characterization of CTC which may enable development of personalized therapeutics against emergent mutations. This warrants further exploration in larger studies to corroborate these preliminary findings, investigate pharmacokinetics, identify biomarkers and should prompt further investigations to develop specific nuclear LSD1 inhibitors.

## Data Availability Statement

The original contributions presented in the study are included in the article/**Supplementary Material**. Further inquiries can be directed to the corresponding author.

## Ethics Statement

The studies involving human participants were reviewed and approved by ACT Human Research Ethics Committee (ETH.10.16.218). The patients/participants provided their written informed consent to participate in this study.

## Author Contributions

TP, conceptualization, methodology, investigation, writing the original draft, writing-review and editing. LM, conceptualization, methodology, investigation, writing-review and editing. TP and LM are joint first authors. RM, WT, AT, and PL, methodology, investigation, writing-review and editing. FW, methodology, software, validation, investigation writing-review and editing. JED: Methodology, investigation, writing-review and editing. PC and EM, data curation, writing-review and editing. DF, formal analysis, writing-review and editing. JC, conceptualization, methodology, funding acquisition, supervision, resources, writing-review, project administration and editing. SR: Led the biomarker analysis for the trial and her lab developed all of the liquid biopsy based assays and correspondence for this aspect should be directed to her. Resources, investigation, validation, conceptualization, supervision, writing–review & editing. DY, resources, supervision, validation, conceptualization, methodology, writing-review, editing. All authors contributed to the article and approved the submitted version.

## Funding

EpiAxis Therapeutics was the Sponsor and provided funding for this trial. EpiAxis funded TP to attend the Medical Oncology Group of Australia ACORD Protocol Development Workshop in 2016. This work was also supported by the National Health and Medical Research Council (Grant ID APP1068065 and GNT1105747) (CIA SR) and the UC Foundation.

## Conflict of Interest

In accordance with NHMRC policy and our ethical obligations as researchers, we report that SR, WT, RM, AT, PL, JED, and FW have equity in EpiAxis Therapeutics Pty Ltd. DY has been an Advisory Board member for Specialised Therapeutics. JC is the CEO of EpiAxis Therapeutics. DF is the Chair of EpiAxis Therapeutics.

The remaining authors declare that the research was conducted in the absence of any commercial or financial relationships that could be construed as a potential conflict of interest.

The authors declare that this study received funding from EpiAxis Therapeutics. The funder had the following involvement with the study: the company instigated the study protocol, data collection, analysis, and interpretation.

## Publisher’s Note

All claims expressed in this article are solely those of the authors and do not necessarily represent those of their affiliated organizations, or those of the publisher, the editors and the reviewers. Any product that may be evaluated in this article, or claim that may be made by its manufacturer, is not guaranteed or endorsed by the publisher.

## Supplementary Material

Supplementary Figure 1 CTC enumeration during each visit.

Supplementary Table 1 Inclusion Exclusion Criteria.

Supplementary Table 2 Definition of DLT
