# Supplementary figures and images for: A Phase 1 Proof of Concept Study Evaluating the Addition of an LSD1 Inhibitor to Nab-Paclitaxel in Advanced or Metastatic Breast Cancer (EPI-PRIMED)
Source: Front Oncol. 2022 Jun 3;12:862427. doi: 10.3389/fonc.2022.862427 (PMC9205212; doi:10.3389/fonc.2022.862427)

**Figure S1.** CTC enumeration during each visit.

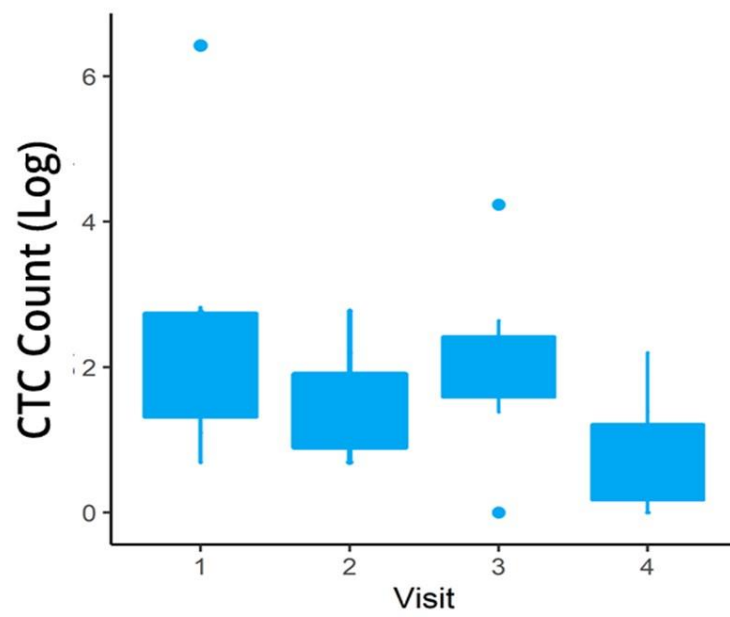

Supplement: Supplementary Figure 1 — CTC enumeration during each visit. [file DataSheet_1.pdf]
