# Supplementary material for: A Phase 1 Proof of Concept Study Evaluating the Addition of an LSD1 Inhibitor to Nab-Paclitaxel in Advanced or Metastatic Breast Cancer (EPI-PRIMED)
Source: Front Oncol. 2022 Jun 3;12:862427. doi: 10.3389/fonc.2022.862427 (PMC9205212; doi:10.3389/fonc.2022.862427)
Supplement: Supplementary Table 1 — Inclusion Exclusion Criteria. [file Table_1.docx]

**Table S1** Inclusion Exclusion Criteria

| Inclusion Criteria  1. Patients who are 18 years or older;  2. Able to understand written and spoken English and in a position to provide written informed consent to participate;  3. A patient who is in a position to attend a 12-week study treatment regimen and end of study visit;  4. Metastatic Breast Cancer (MBC) or inoperable locally advanced breast cancer diagnosis based on pre-existing documented histopathology and medical imaging results as well as other validated immunopathology testing where necessary, either TNBC or not (Cohorts A-E);  5. Women with metastatic breast cancer or inoperable locally advanced breast cancer who have not received any cytotoxic therapy in the last 3 weeks;  6. Volunteers of child-bearing potential must have a negative serum pregnancy test (serum beta-human chorionic gonadotropin or β-hCG) and have agreed to practice effective, reliable contraceptive regimen as described in Section 11.3.4 for the duration of this clinical trial;  7. ECOG Performance Status 0 or 1; and  8. Adequate liver function as evidenced by bilirubin of <1.5 times upper limit of normal (ULN) and ALT/AST <2 times of ULN. However, AST and ALT of <5 times ULN is acceptable if liver metastases are present.  Exclusion Criteria  1. A patient who has been diagnosed as having HER2-positive metastatic breast cancer;  2. A concurrent condition that may limit the decision-making capabilities of the participant during the informed consent process;  A previous positive diagnosis of Human Immunodeficiency Virus (HIV) and/or Hepatitis C Virus (HCV) and/or Hepatitis B Virus (HBV) infection;  4. Women who are pregnant or lactating;  5. Uncontrolled, untreated intra-cranial metastasis. However, controlled intra-cranial metastasis are allowed, i.e. stable patients with, more than a month after the completion of whole brain radiotherapy and not currently on steroids or anticonvulsants;  6. Current use of monoamine oxidase inhibitors (MOAI) or use of dextromethorphan; 7. Current use of CNS depressants such as selective serotonin re-uptake inhibitors as well as specific medication for pain management that include pethidine, tramadol, dextromethorphan, fentanyl and/or methadone. This includes the concurrent use of any serotoninergic agents or Buspirone Hydrochloride during the week preceding phenelzine administration, the active study treatment phase and the washout period at the end of study. Serotoninergic drugs may include but are not limited to the following: dexfenfluramine, fluoxetine, fluvoxamine, paroxetine, sertraline, citalopram and venlafaxine;  8. Previous use of Abraxane. Prior use of Abraxane in an adjuvant setting may be acceptable, at the discretion of the Sponsor, while Abraxane prescribed for metastatic disease is acceptable only if Abraxane was not administered during the 3 years prior to enrolment;  9. Known allergy to phenelzine sulfate or similar MOAI;  10. Known or suspected history of alcohol abuse;  11. Other clinically significant medical conditions including but not limited to cardiovascular, neurological, psychiatric, renal, hepatic, haematological or endocrine abnormalities that in the PI’s opinion are uncontrolled and likely to affect the volunteer’s ability to comply with the study requirements;  12. Persistent uncontrolled systemic hypertension (> 180/110 mmHg) and/ or the use of guanethidine;  13. Clinically significant dysrhythmia detected from the screening visit ECG report, including left bundle branch block. That is, arrhythmias that are considered to be related to ongoing electrical conduction pathology and not related to treatment circumstance and self- limiting such as sinus arrhythmias. Controlled atrial fibrillation (for example a ventricular rate of ≤ 90 beats per minute, or any other AF which is judged by the PI to be controlled) will not be considered an exclusion factor;  14. Clinically significant anaemia (haemoglobin <90g/L) from the screening visit haematology report;  15. Severe renal impairment where creatinine clearance < 30mL/minute at the time of the screening visit based on clinical chemistry results;  16. Major elective surgery scheduled during the course of active treatment administration and follow up;  17. Social factors that are likely to affect the patient’s ability to comply with the requirements of the study protocol;  18. Concurrent enrolment in another interventional (medical or surgical) clinical trial during the course of this clinical trial (between screening and End of Study (inclusive));  19. Evidence of recreational methamphetamine and/ or cocaine use as confirmed by a drugs of abuse screen;  20. Concurrent use of sympathomimetic drugs contraindicated with phenelzine including methylphenidate, dopamine, epinephrine, and/or norepinephrine or related compounds such as methyldopa, L-dopa, L-tryptophan, L-tyrosine, and/or L-phenylalanine during this study or less than 2 weeks prior to the baseline visit;  21. Current radiation therapy other than palliative treatment to a painful peripheral site; and  22. Patients with a life expectancy < 3 months. |
| --- |
