# Supplementary material for: A Phase 1 Proof of Concept Study Evaluating the Addition of an LSD1 Inhibitor to Nab-Paclitaxel in Advanced or Metastatic Breast Cancer (EPI-PRIMED)
Source: Front Oncol. 2022 Jun 3;12:862427. doi: 10.3389/fonc.2022.862427 (PMC9205212; doi:10.3389/fonc.2022.862427)
Supplement: Supplementary Table 2 — Definition of DLT [file Table_2.docx]

**Supplementary Table S2**: Definition of DLT

| For this study, a DLT was defined as an adverse event or abnormal laboratory value assessed as unrelated to disease, disease progression, inter-current illness, or concomitant medications that occurs between Day 2 and 56 (inclusive) of study treatment and meets any of the criteria included as below.   - Grade ≥2 peripheral neuropathy - Grade 3 Febrile neutropenia (Grade 3 neutropenia associated with fever ≥ 38.5°C); - Any Grade 3 non-haematological toxicity attributable to the study treatments except alopecia; nausea, vomiting, or diarrhoea for 72 hours because of inadequate use of prophylaxis; - Grade 3 fatigue (asthenia) for > 7 days; - Non-hematologic Grade 3 or 4 laboratory adverse event that do not return to baseline or to Grade 1 within 7 days, and which is judged clinically significant by the PI. That is, at the discretion of the PI some Grade 3 or Grade 4 non haematologic AEs which fail to return to baseline or grade 1 within 7 days may be not be considered clinically significant and may not constitute a DLTs; - Grade 3 thrombocytopenia with signs of significant bleeding or platelet count Grade 4; - Blood bilirubin (total bilirubin) Grade ≥3 for 72 hrs, AST or ALT Grade 3 for >7 consecutive days, AST or ALT Grade 4; - Persistent grade 3 hypertension for >7 days and not responding to antihypertensive therapy or grade 4 hypertension; - An inability to administer treatment (with >7day delay) on Days 8 and 15 during Cycle 1 and during the equivalent in Cycle 2 for toxicity reason; and - Any other treatment emergent SAE that are considered to be unexpected and results in a sequelae that is a direct cause of the study intervention and thus require patient withdrawn by the treating physician. |
| --- |
